# Outcomes of Patients Hospitalized for Acute Diverticulitis With Comorbid Generalized Anxiety Disorder

**DOI:** 10.7759/cureus.35461

**Published:** 2023-02-25

**Authors:** Alexander J Kaye, Shivani J Patel, Sarah R Meyers, Pooja Saiganesh, Sushil Ahlawat

**Affiliations:** 1 Internal Medicine, Rutgers University New Jersey Medical School, Newark, USA; 2 Psychiatry, Rutgers Robert Wood Johnson Medical School, Piscataway, USA; 3 Gastroenterology and Hepatology, State University of New York Downstate Health Sciences University, Brooklyn, USA

**Keywords:** acute respiratory failure, hypotension, intestinal abscess, intestinal obstruction, generalized anxiety disorder (gad), diverticulitis

## Abstract

Introduction

Diverticular disease and anxiety disorders are common in the general population. Prior research on diverticular disease showed that these patients have an increased frequency of anxiety and depression. The objective of this study was to explore the impact of generalized anxiety disorder (GAD) on the outcomes of adult patients admitted with acute diverticulitis.

Methods

Using the National Inpatient Sample database from the year 2014 and International Classification of Diseases, Ninth Edition Revision, Clinical Modification (ICD-9 CM) codes, acute diverticulitis patients were selected. The outcomes of diverticulitis patients with and without GAD were explored. The outcomes of interest included inpatient mortality, hypotension/shock, acute respiratory failure, acute hepatic failure, sepsis, intestinal abscess, intestinal obstruction, myocardial infarction, acute renal failure, and colectomy. A multivariate logistic regression analysis was performed to determine if GAD is an independent predictor for the outcomes.

Results

Among 77,520 diverticulitis patients in the study, 8,484 had comorbid GAD. GAD was identified as a risk factor for intestinal obstruction (adjusted odds ratio (aOR) 1.22, 95% CI: 1.05-1.43, p<0.05), and intestinal abscess (aOR 1.19, 95% CI: 1.10-1.29, p<0.05). GAD was found to be a protective factor for hypotension/shock (aOR 0.83, 95% CI: 0.76-0.91, p<0.05) and acute respiratory failure (aOR 0.76, 95% CI: 0.62-0.93, p<0.05). The aORs of sepsis, inpatient mortality, myocardial infarction, acute renal failure, and colectomy were not statistically significant.

Conclusions

Patients with acute diverticulitis who are also diagnosed with GAD are at increased risk for intestinal obstruction and intestinal abscess, which may be due to the influence GAD has on the gut microbiota as well as the impact of GAD pharmacotherapy on gut motility. There was also a decreased risk for acute respiratory failure and hypotension/shock appreciated in the GAD cohort which may be attributable to the elevated healthcare resource utilization seen generally in GAD patients, which may allow for presentation to the emergency department, hospitalization, and treatment earlier in the diverticulitis disease course.

## Introduction

During colonoscopy, diverticulosis is a frequent finding in adult patients. The prevalence of diverticulosis is as high as ~50% among patients above the age of 60 years [1]. Approximately 10% to 25% of patients with diverticulosis develop a symptomatic disease during their lifetime. There are multiple non-modifiable risk factors for diverticulosis, which include advanced age and some genetic abnormalities such as Ehlers-Danlos and renal polycystic disease [2-4]. A particularly common non-modifiable risk factor for diverticulosis is the male sex [2]. Possible modifiable risk factors include elevated body mass index, smoking, and medication use including steroids, non-steroidal anti-inflammatory drugs, and opiates [2,5-8]. Less well-studied possible risk factors for diverticulosis include colonic dysmotility due to neuronal degeneration and altered colonic neuromuscular activity due to changes in serotonin signaling [2,9,10]. Complications of diverticular disease can include acute diverticulitis, abscess, fistula, bowel obstruction, and perforation.

Prior studies demonstrated that a diagnosis of diverticular disease is associated with an elevated frequency of anxiety and depressive disorders [11]. Notably, anxiety and major depressive disorders are relatively well studied regarding their relationship with inflammatory bowel disease (IBD) and irritable bowel syndrome (IBS) with these patients having more severe disease and acute flares [12,13]. Generalized anxiety disorder (GAD) is considered a common psychiatric diagnosis with a lifetime prevalence of 4.6% and 7.7% in male and female patients of ages 18-64 years, respectively [14]. The specific pathophysiology of GAD is unclear; however, there is thought to be an association with the serotonin and noradrenergic systems [15].

Notably, while anxiety disorders are associated with worse outcomes in IBD and IBS patients, standard pharmacologic treatment for anxiety disorders can also impact the gastrointestinal tract. Pharmacologic treatment of GAD includes serotonin-norepinephrine reuptake inhibitors (SNRIs) and selective serotonin reuptake inhibitors (SSRIs) as first-line therapeutic interventions, with second-line agents including buspirone, benzodiazepines, and pregabalin and second-generation antipsychotics. While these therapeutics are intended to alleviate anxiety, several of these medications have known gastrointestinal adverse effects. In particular, SSRIs and SNRIs are theorized to affect gut motility due to their impact on serotonin receptors and serotonin levels [16]. The gastrointestinal side effect profile for SSRIs and SNRIs includes nausea, vomiting, diarrhea, and weight changes. Patients using SSRIs have also been found to be at increased risk of irritable bowel syndrome [17].

Despite the link between diverticular disease and anxiety disorders, there has been little research about the impact anxiety has on diverticulitis. Therefore, we aimed to identify the clinical outcomes of patients admitted for diverticulitis who also have comorbid GAD.

This research was previously presented as a poster at the Annual American College of Gastroenterology conference on October 24, 2022.

## Materials and methods

A retrospective cohort study was performed for all adult patients (defined as patients 18 years old and older) who were hospitalized due to diverticulitis in the year 2014. Institutional review board approval was not required for this research project in light of no patient-level data being utilized. The data were extracted from the National Inpatient Sample (NIS), a database developed for the Healthcare Cost and Utilization Project, which is sponsored by the Agency for Healthcare Research and Quality [18]. The NIS database is widely recognized as the biggest all-payer inpatient database in the United States of America. The International Classification of Diseases, Ninth Edition Revision, Clinical Modification (ICD-9 CM) codes were utilized to identify all of the diagnoses from the NIS database. The patients included in this study were stratified into two groups: those with a history of GAD and those who lack a history of GAD. Between these two groups, demographic information and data about their hospitalization including age, sex, race, length of stay, and hospitalization cost were extracted and subsequently compared. The Charlson comorbidity index, which is an established tool that is used to adjust for confounding variables, was also compared between these groups [19,20].

All of the statistical analyses were performed using Windows, Version 28 (Released 2021; IBM Corp., Armonk, New York, United States). The outcomes of interest collected for these two groups were hypotension/shock, myocardial infarction, acute renal failure, acute respiratory failure, acute hepatic failure, sepsis, intestinal abscess, intestinal obstruction, colectomy, and inpatient mortality. These outcomes were then compared between these groups. Means and proportions were compared using independent T-tests and chi-squared tests, respectively. The statistical analyses performed were two-tailed, with a p-value threshold of under 0.05 being considered statistically significant. Categorical variables were described as numbers (N) and percentages (%), while continuous variables were reported as means ± standard deviation (SD). A multivariate logistic regression analysis was also conducted to establish whether GAD is an independent predictor of the aforementioned outcomes, after age, sex race, and Charlson comorbidity index had been adjusted for.

## Results

During the 2014 year, 77,520 adults were hospitalized due to diverticulitis. Among these diverticulitis patients, 8,484 of them had a history of GAD. As seen in Table 1, this subgroup of diverticulitis patients with GAD were younger (62.68 years old vs. 63.24 years old, p < 0.05), more likely to be female (72.6% vs. 43.5%, p < 0.05), more likely to be Caucasian (83.9% vs. 75.5%, p < 0.05), and had a longer length of stay (4.86 days vs. 4.53 days, p < 0.05). No statistically significant differences were identified in the Charlson comorbidity index (2.89 with GAD vs. 2.85 without, p = 0.15) and total hospital charge ($40,003.19 with GAD vs. $39,659.51 without, p = 0.54).

**Table 1:** Demographics, characteristics, length of stay, total hospital charge, and Charlson comorbidity index among diverticulitis patients with and without a history of generalized anxiety disorder

	With generalized anxiety disorder	Without generalized anxiety disorder	p-value
N = 77,520	N = 8,484	N = 69,036	
Patient age, mean (SD)	62.68 (14.62)	63.24 (15.63)	<0.05
Sex, N (%)			<0.05
Female	6,160 (72.6%)	30,033 (43.5%)	
Male	2,321 (27.4%)	38,973 (56.5%)	
Race, N (%)			<0.05
White	6,836 (83.9%)	49,907 (75.5%)	
Black	429 (5.3%)	6,292 (9.5%)	
Hispanic	678 (8.3%)	7,217 (10.9%)	
Asian or Pacific Islander	32 (0.4%)	907 (1.4%)	
Native American	21 (0.3%)	271 (0.4%)	
Other	148 (1.8%)	1,542 (2.3%)	
Length of stay, in days (SD)	4.86 (4.49)	4.53 (4.45)	<0.05
Total hospital charges, in $ (SD)	40,003.19 (46,843.99)	39,659.51 (53,898.20)	0.54
Charlson comorbidity index (SD)	2.89 (0.02)	2.85 (2.23)	0.15

In Table 2, the outcomes of diverticulitis patients with and without comorbid GAD were compared. The diverticulitis patients with a history of GAD had an increased likelihood of acute respiratory failure (1.4% vs. 1.1%, p < 0.05), and hypotension/shock (7.1% vs. 5.9%, p < 0.05). Diverticulitis patients without a history of GAD were more likely to have an intestinal abscess (10.7% vs. 9.0%, p < 0.05), colectomy (2.8% vs. 2.2%, p < 0.05), sepsis (5.6% vs. 5.1%, p < 0.05), and had a higher inpatient mortality (0.6% vs. 0.4%, p < 0.05). No statistically significant difference in intestinal obstruction (p = 0.12), acute renal failure (p = 0.08), and myocardial infarction (p = 0.39) was found between diverticulitis patients with and without comorbid GAD. Because of the small sample size for acute hepatic failure, further analysis of this outcome could not be performed.

**Table 2:** Unadjusted clinical outcomes among diverticulitis patients with and without a history of generalized anxiety disorder *Exact number is not included in the table due to small sample size (10 patients or fewer)

Outcomes	With generalized anxiety disorder	Without generalized anxiety disorder	p-value
Intestinal obstruction	51 (0.6%)	329 (0.5%)	0.12
Intestinal abscess	761 (9.0%)	7,354 (10.7%)	<0.05
Colectomy	190 (2.2%)	1,932 (2.8%)	<0.05
Sepsis	430 (5.1%)	3,871 (5.6%)	<0.05
Acute hepatic failure	*	66 (0.1%)	0.97
Acute respiratory failure	119 (1.4%)	765 (1.1%)	<0.05
Acute renal failure	593 (7.0%)	5,186 (7.5%)	0.08
Myocardial infarction	55 (0.6%)	506 (0.7%)	0.39
Hypotension/shock	600 (7.1%)	4,057 (5.9%)	<0.05
Inpatient mortality	33 (0.4%)	398 (0.6%)	<0.05

The adjusted odds ratios (aORs) of the different outcomes, after controlling for the Charlson comorbidity index, race, sex, and age, are displayed in Table 3. GAD was subsequently identified as an independent risk factor for intestinal abscess (aOR 1.19, 95% confidence interval (CI): 1.10-1.29, p < 0.05) and intestinal obstruction (aOR 1.22, 95% CI: 1.05-1.43, p < 0.05). In addition, GAD was found to be a protective factor for acute respiratory failure (aOR 0.76, 95% CI: 0.62-0.93, p < 0.05), and hypotension/shock (aOR 0.83, 95% CI: 0.76-0.91, p < 0.05). The p-values for the aORs of sepsis (aOR 1.07, 95% CI: 0.97-1.19, p = 0.19), inpatient mortality (aOR 1.34, 95% CI: 0.93-1.92, p = 0.11), myocardial infarction (aOR 1.05, 95% CI: 0.78-1.40, p = 0.77), acute renal failure (aOR 1.02, 95% CI: 0.93-1.11, p = 0.76), and colectomy (aOR 0.75, 95% CI: 0.55-1.02, p = 0.07) did not meet the cutoff for statistical significance.

**Table 3:** Multivariate logistic regression analysis of clinical outcomes among diverticulitis patients with and without a history of generalized anxiety disorder *Adjusted for age, sex, race, and the Charlson comorbidity index

Outcomes	Adjusted odds ratio*	95% Confidence interval	p-value
Intestinal obstruction	1.22	1.05-1.43	<0.05
Intestinal abscess	1.19	1.10-1.29	<0.05
Colectomy	0.75	0.55-1.02	0.07
Sepsis	1.07	0.97-1.19	0.19
Acute respiratory failure	0.76	0.62-0.93	<0.05
Acute renal failure	1.02	0.93-1.11	0.76
Myocardial infarction	1.05	0.78-1.40	0.77
Hypotension/shock	0.83	0.76-0.91	<0.05
Inpatient mortality	1.34	0.93-1.92	0.11

While Table 2 and Table 3 outline the same outcomes, the data initially appear to be in conflict. For example, in Table 2, intestinal abscess is seen to occur less commonly in the GAD group with a statistically significant p-value. In comparison, Table 3 demonstrates this same outcome occurring more commonly in the GAD group with a statistically significant p-value. This difference can be explained by Table 3 displaying data that have been adjusted for many potential confounding factors.

## Discussion

Previous studies showed that diverticular disease is associated with an elevated prevalence of anxiety and depressive disorders [12,13]. Despite this association, the impact anxiety has on medical outcomes of diverticulitis was unclear. This study is the first to investigate the outcomes of diverticulitis among inpatients with comorbid GAD. The findings in this study demonstrated that patients hospitalized due to diverticulitis who have comorbid GAD have an increased risk of intestinal abscess and intestinal obstruction. This represents a critical finding due to the increased mortality risk associated with intestinal obstruction and intestinal abscess [21,22]. These findings may be attributable in part to the underlying pathophysiology of GAD and the impact of the pharmacologic interventions used for GAD. Serotonin signaling in the enteric tract can modulate colonic peristalsis [23]. Close to 95% of the body’s production of serotonin is attributed to the gastrointestinal epithelium [24]. Patients with GAD are noted to have an altered serotonergic signaling in the central nervous system and the use of SSRIs can increase the synaptic level of monoamines and lead to more activation of postsynaptic receptors [24]. Patients with diverticulitis have been observed to have a significant decrease in SERT (a serotonin transporter) transcript levels and have changes in attenuation of SERT expression and function [10]. Diverticular disease, possibly as a consequence, has also been associated with an increased number of serotonin-producing cells in the colonic mucosa [25]. Chronic SSRI and SNRI use may upregulate colonic serotonin signaling leading to increased colonic phasic contractility, which affects colonic motility [26]. Changes in colonic motility can lead to dysmotility, which may contribute to the increased risk of intestinal obstruction and intestinal abscess in diverticulitis patients with comorbid GAD.

The other possible pathophysiology leading to increased complications of intestinal obstruction and abscess in patients with diverticulitis and GAD is dysregulation of the gut-brain axis. Animal models for depression have been seen to have altered enteric microbiota [24]. A study on patients with GAD noted that these patients also have altered enteric microbiota as compared to those without GAD [27]. While the relationship between enteric microbiota and acute diverticulitis requires further investigation, early studies show that elevated levels of certain intestinal bacteria, such as *Subdoligranulum* species and *Marvinbryantia* species, can lead to inflammation [28,29]. This increased colonic inflammation may possibly contribute to the elevated risk of intestinal obstruction and abscess in the GAD cohort [28].

Our study found that GAD was also associated with a decreased likelihood of acute respiratory failure and hypotension/shock. These outcomes may be less likely as a result of an earlier diagnosis and treatment of diverticulitis. In prior studies, patients who have comorbid GAD as well as other anxiety disorders were noted to have higher healthcare utilization, including primary care, emergency department visits, and hospitalizations [30]. Therefore, GAD patients may seek out medical care earlier and more frequently if they experience symptoms of diverticulitis, allowing intervention earlier in the disease process and decreasing the likelihood of progression to acute respiratory failure and hypotension/shock.

There are several limitations to this study. One limitation of this study relates to the process of conducting research using the NIS database, which depends on billing codes input by medical providers, which may lack precision. Inaccurate billing code use may result in over or underrepresentation of the subgroup of diverticulitis patients with GAD. In addition, due to lack of ICD-9 coding for SSRI and SNRI use, there was no way to explore which patients were taking anti-anxiety medications at the time of their hospitalization. Another limiting factor is that the NIS database only includes hospitalized patients. Therefore, any acute diverticulitis patients cared for exclusively as outpatients were not captured. While this study has these limitations, a strength of this study is the ability to assess patient demographics and outcomes on a national scale. This research was also strengthened by the utilization of the multivariate logistic regression analysis, which was able to adjust for possible confounding variables.

## Conclusions

In conclusion, acute diverticulitis patients with comorbid GAD had a higher risk of intestinal abscess and intestinal obstruction and a decreased risk for acute respiratory failure and hypotension/shock. Given that patients with GAD who present to the hospital with acute diverticulitis are more likely to have intestinal obstruction and abscess, providers should consider having a lower threshold to work up or start treatment for these pathologies as both are associated with elevated mortality. Further investigation into whether well-controlled GAD has improved outcomes in acute diverticulitis as compared to poorly controlled GAD would provide important information on inpatient healthcare providers in terms of risk stratification decisions in the hospital as well as guidance to outpatient providers about the urgency to initiate treatment for GAD patients who have known diverticulosis.
